# Naphthol as an Antiseptic

**Published:** 1888-03

**Authors:** 


### Naphthol as an Antiseptic.

M. Bouchard, in *Comptes Rend.*, gives his experience with this substance and claims that Beta naphthol is absolutely safe in any form it is likely to be administered. Experiments on animals prove conclusively that it would require nearly one-half pound of it taken internally to produce fatal results on a healthy person weighing one hundred and fifty pounds. When injected into the veins of animals as a one per cent. solution, the amount to produce fatality was equal to 400 grains for an adult of one hundred and fifty pounds. Ten grains in a quart of water will prevent the growth of any organisms. It has five times the antiseptic power of carbolic acid, four times that of creosote, three times that of iodoform, five times that of iodol, four times that of naphtaline, but has only $\frac{1}{20}$ the destructive energy of biniodide of mercury.—*Pharmaceutical Record.*

Photographic Diagnosis.—The following fact may suggest a new medical use for photography. A lady, on whose face no spot or pimple was discernible, was lately photographed. On examining the proof the photograher was surprised to find the face was covered with black specs. A second photograph was taken, with the same results. The following day the lady died of smallpox. The superior sensitiveness of the plate revealed what was invisible to the naked eye.

